# CaMKIIδB Mediates Aberrant NCX1 Expression and the Imbalance of NCX1/SERCA in Transverse Aortic Constriction-Induced Failing Heart

**DOI:** 10.1371/journal.pone.0024724

**Published:** 2011-09-13

**Authors:** Ying-Mei Lu, Jiyun Huang, Norifumi Shioda, Kohji Fukunaga, Yasufumi Shirasaki, Xiao-ming Li, Feng Han

**Affiliations:** 1 Institute of Pharmacology, Toxicology and Biochemical Pharmaceutics, Zhejiang University, Hangzhou, China; 2 Department of Neurobiology, Zhejiang University School of Medicine, Hangzhou, China; 3 Department of Pharmacology, Graduate School of Pharmaceutical Sciences, Tohoku University, Sendai, Japan; 4 Biological Research Laboratories, Daiichi-Sankyo Pharmaceutical Co., Ltd. Tokyo, Japan; Yale Medical School, United States of America

## Abstract

Ca^2+^/calmodulin-dependent protein kinase II δB (CaMKIIδB) is one of the predominant isoforms of CaMKII in the heart. The precise role of CaMKIIδB in the transcriptional cross-talk of Ca^2+^-handling proteins during heart failure remains unclear. In this work, we aim to determine the mechanism of CaMKIIδB in modulating the expression of sarcolemmal Na^+^–Ca^2+^ exchange (NCX1). We also aim to address the potential effects of calmodulin antagonism on the imbalance of NCX1 and sarcoendoplasmic reticulum Ca^2+^ ATPase (SERCA) during heart failure. Eight weeks after transverse aortic constriction (TAC)-induced heart failure in mice, we found that the heart weight/tibia length (HW/TL) ratio and the lung weight/body weight (LW/BW) ratio increased by 59% and 133%, respectively. We further found that the left ventricle-shortening fraction decreased by 40% compared with the sham-operated controls. Immunoblotting revealed that the phosphorylation of CaMKIIδB significantly increased 8 weeks after TAC-induced heart failure. NCX1 protein levels were also elevated, whereas SERCA2 protein levels decreased in the same animal model. Moreover, transfection of active CaMKIIδB significantly increased NCX1 protein levels in adult mouse cardiomyocytes *via* class IIa histone deacetylase (HDAC)/myocyte enhancer factor-2 (MEF2)-dependent signaling. In addition, pharmacological inhibition of calmodulin/CaMKIIδB activity improved cardiac function in TAC mice, which partially normalized the imbalance between NCX1 and SERCA2. These data identify NCX1 as a cellular target for CaMKIIδB. We also suggest that the CaMKIIδB-induced imbalance between NCX1 and SERCA2 is partially responsible for the disturbance of intracellular Ca^2+^ homeostasis and the pathological process of heart failure.

## Introduction

Ca^2+^/calmodulin-dependent protein kinase II (CaMKII) is involved in the development of cardiac hypertrophy and heart failure. Predominant expression of the CaMKIIδ isoform contributes to cardiac decompensation by enhancing the ryanodine receptor (RyR)-mediated sarcoplasmic reticulum (SR) Ca^2+^ leak, and attenuating CaMKIIδ activation has been shown to limit the progression to heart failure [1]–[2]. CaMKIIδ regulates a number of key proteins, including phospholamban (PLB), RyRs, and the L-type channels, which are involved in intracellular Ca^2+^ homeostasis [1]–[4]. As one of the two primary splicing variants of the δ isoform in the heart of many mammals, CaMKIIδB localizes to the nucleus and is predicted to play a predominant role in Ca^2+^-mediated transcriptional gene regulation. Transient expression of CaMKIIδB induces hypertrophy and ANF production in neonatal rat ventricular myocytes [5]. The over-expression of CaMKIIδB in transgenic mice has been shown to induce hypertrophy-related gene expression and result in cardiac hypertrophy [5], [6]. However, little is known about the relationship between aberrant CaMKIIδB expression and the cross talk of Ca^2+^-handling proteins during heart failure.

In the physiological context, the depolarization of an action potential activates L-type Ca^2+^ channels and increases Ca^2+^ influx in the heart. This triggers the release of more Ca^2+^ from the sarcoendoplasmic reticulum Ca^2+^-ATPase (SERCA2) *via* RyRs, an event typically referred to as Ca^2+^-induced Ca^2+^ release. During relaxation, Ca^2+^ is immediately transported into the SR *via* SERCA2 and extruded by sarcolemmal Na^+^–Ca^2+^ exchange (NCX1) in cardiomyocytes [7]. Features of a failing heart include a prolonged action potential and depressed contractility, because Ca^2+^ overload has substantial, adverse effects in failing hearts. Several investigators have shown that during heart failure, the expression and activity of the Ca^2+^-sequestering SERCA2 is decreased, RyR is hyperphosphorylated, and/or activity and protein levels of NCX1 are increased [8]–[11]. A study by Andersson *et al.* showed that inducible cardiomyocyte-specific excision of the *Serca2* gene leads to a substantial reduction in diastolic function in mice [12]. Over-expression of SERCA2a improves the contraction performance of cardiomyocytes in adrenergically stimulated adult rats [13]. As another essential regulator of Ca^2+^ homeostasis, NCX1 is reported to be up-regulated at the transcriptional level during cardiac hypertrophy, ischemia, and failure [9], [14]. Meanwhile, Muller *et al*. demonstrated that the feline *1831Ncx1* H1 promoter is sufficient for the up-regulation of *Ncx1* in response to pressure overload in transgenic mice [15]. These observations suggest that reduced SERCA function and enhanced NCX1 function are associated with cardiac dysfunction in mammalian heart failure. However, the question arises as to whether CaMKIIδB can modulate the transcriptional cross-talk and balance the expression of Ca^2+^-handling proteins in a failing heart. Accordingly, we tested the hypothesis that heart failure induced by transverse aortic constriction (TAC) is associated with an increase in CaMKIIδB activity, an imbalance of Ca^2+^-handling proteins and altered Ca^2+^ homeostasis.

To better understand the molecular basis of aberrant Ca^2+^ handling during heart failure, we sought to determine the potential role of CaMKIIδB in modulating both the expression of NCX1 and NCX1/SERCA2 balance during the pathological process of heart failure. Here, we demonstrate a correlation between the activation of CaMKIIδB and the elevation of NCX1 protein levels during TAC-induced heart failure in mice. The over-expression of CaMKIIδB led to an increase in NCX1 expression and a disturbance of the NCX1/SERCA2 balance *via* class IIa histone deacetylase (HDACs)/myocyte enhancer factor-2 (MEF2)-dependent signaling. Moreover, treatment with a calmodulin antagonist improved cardiac function and normalized the NCX1/SERCA2 balance away from heart failure. These findings not only define a novel function for CaMKIIδB in Ca^2+^-handling proteins but also highlight the potential therapeutic benefits of a novel calmodulin antagonist against heart failure.

## Materials and Methods

### Animals

Adult male DDY mice weighing 35–40 g were obtained from Nippon SLC (Hamamatsu, Japan). Eight-week-old males were acclimated to the local environment for 1 week, which included housing in polypropylene cages at 23±1°C in a humidity-controlled environment maintained on a 12-h light–dark schedule (lights on 8:00 a.m. – 8:00 p.m.). Mice were provided food and water *ad libitum*. All the animal experiments were carried out in accordance with the National Institute of Health guidance for the care and use of laboratory animals, were approved by The Committees for Animal Experiments in Tohoku University in Japan (21–252) and Zhejiang University in China (SCXK 2007–0029).

### Surgical procedure and drug treatment

Transverse aortic constriction (TAC) was performed on mice as described previously [16]–[17]. Two weeks after the TAC or sham surgery, mice were orally administered either a vehicle or DY-9836 (10 or 20 mg/kg) daily for another 6 weeks. Mice were decapitated 8 weeks after TAC, and the heart was dissected out for further analysis. In addition, the mortality rate of TAC mice was addressed using Kaplan-Meier survival plots.

### Histological examination

Mouse heart samples were cut into transverse sections and stained with hematoxylin and eosin (H&E) as described previously [16]. Cross-sectional images of cardiomyocytes were scanned at 400× magnification to evaluate the extent of cardiomyocyte hypertrophy.

### Measurement of cardiac hypertrophy

After 6 weeks of drug administration (DY-9836, 20 mg/kg), the mice were sacrificed by cervical dislocation; then, the hearts and lungs were removed and weighed as previously described [16]. Relative heart weights (HW), tibia length (TL) and lung weights (LW) were calculated as ratios with body weight (HW/TL and LW/BW), and cardiac geometry (wall thickness and chamber diameter) was determined, which were used to estimate the degree of cardiac hypertrophy.

### Transthoracic echocardiography

Non-invasive echocardiographic measurements were performed using ultrasonic diagnostic equipment (Aloka, SSD-6500, Tokyo, Japan) as described previously [16].

### Western blot analysis

All animals were sacrificed after 6 weeks of drug administration, and their hearts were immediately harvested and stored at −80°C until immunoblotting analyses were performed, as described previously [17]. The following antibodies were used: NCX1 mouse monoclonal antibody and MEF2 rabbit polyclonal antibody (1∶1000, Santa Cruz Biotechnology, Inc.), phospho-phospholamban (Thr-17; 1∶1000, Santa Cruz Biotechology, Santa Cruz, CA, and Ser-16; 1∶1000, Upstate, Lake Placid, NY), SERCA2 mouse monoclonal antibody (1∶1000, Sigma, St. Louis, MO), isoforms 1–4 of CaMKII rabbit polyclonal antibody (1∶500, Trans Genic Inc.), HDAC4 (1∶1000, Abcam, Cambridge, UK), active caspase-3 polyclonal antibody (1∶500, Cell Signaling Technology) and β-tubulin antibody (1∶10000, Sigma, St. Louis, MO).

### Adult cardiomyocyte cell culture and small interfering RNA transfection

Adult mice cardiomyocytes were isolated as described previously [18]. Adult cardiomyocytes were transfected with activated CaMKIIδB (T278D), dominant negative CaMKIIδB (K43E), MEF2c siRNA, or control siRNA, as described previously [19]. After 24 h, a new serum-free DMEM supplement with endothelin-1 (ET, 100 nM) was added, and the cells were incubated for another 24 h.

### Immunohistochemistry studies

Confocal immunocytochemical imaging was performed as previously described [16]. For immunolabeling, sections were incubated with a CaMKII 1–4 rabbit polyclonal antibody (1∶100); troponinT and HDAC4 mouse monoclonal antibody (1∶100, Abcam, Cambridge, UK); NCX1 mouse monoclonal antibody (1∶100, Santa Cruz Biotechnology, Inc.); α-actinin and active caspase-3 polyclonal antibody (1∶200, Cell Signaling Technology). To label the cell nuclei (blue), 4′-6-Diamidino-2-phenylindole (DAPI, Sigma, St. Louis, MO) and TO-PRO3 (Invitrogen) was used as a counterstain.

### Chromatin immoprecipitation (ChIP)

ChIP assay was performed on MEF2 in the *NCX1* promoter region using a ChIP kit (Pierce, Thermo Scientific, Rockford, IL) according to manufacturers' protocol. Briefly, cardiac cells were treated with 1% formaldehyde and collected by brief centrifugation, lysed in lysis buffer, and micrococcal nuclease digestion (MNase digestion). Protein-DNA complexes were immunoprecipitated using MEF2 antibody (Santa Cruz Biotechology, Santa Cruz, CA) or IgG as a control. MEF2/DNA formaldehyde cross-linkages were reversed by heating at 65°C for 2 h, DNA purified by DNA Clean-Up Column and resuspended in 50 µl of DNA Column Elution Solution. PCR was performed using 1∶10 dilution of input chromatin (input DNA) or immunoprecipitated chromatin (IP DNA). NCX1 primers sequences were forward 5′- TTCGGCTTTCGCTTCC ATACA-3′ and reverse 5′- CTCTGGCCTTCTGCTTTTCCT-3′. PCR conditions included: an initial 2 min denaturation at 94°C followed by 40 cycles of 94°C for 30 s, 55°C for 30 s and 72°C for 30 s; a final 5 min elongation at 72°C.

### Luciferase assays

Small interfering RNAs (siRNAs) against MEF2 and control siRNA were obtained from Santa Cruz Biotechnology. siRNAs were introduced into cardiomyocytes with transfection medium according to the manufacturer's instructions. After 6 h incubation, the fresh serum-free DMEM was changed, and then cells were transiently transfected with 0.3 µg/well of pGL3-NCX1 reporter construct using Lipofectamine 2000 (Invitrogen). For co-transfections, pRL-TK (Promega) encoding Renilla luciferase (Rluc) served as an internal control. 0.3 µg/well of pGL3-NCX1 reporter construct was transfected along with activated FLAG-tagged CaMKIIδB (CaMKIIδB-T287D). After transfection for 24 h, ET (100 nM) was added to the serum-free DMEM and cultures were incubated for another 24 h. Firefly and Renilla luciferase activities were measured using the Dual-Luciferase Reporter Assay System (Promega) with a luminometer (GENG LIGHT 55, Yamato Scientific Co., Ltd, Tokyo, Japan).

### Measurement of intracellular Ca^2+^ in adult cardiomyocytes using Fura-2AM

Adult mice cardiomyocytes were isolated as described previously [18]. The isolated cells were cultured with 10% FBS in DMEM for 24 h in a 35 mm dish. After incubation overnight, the cells were loaded with Fura-2 AM as described previously [17]. The amplitude of the caffeine-induced Ca**^2+^** transient was used as an index of SR Ca**^2+^** content. Changes in the caffeine-induced Ca^2+^ release from the SR were determined using a ratio of the fluorescence emission at 510 nm in response to excitation at 340 and 380 nm.

### TUNEL assay

*In situ* DNA fragmentation was assessed using a TUNEL assay as previously described [20]. Images were recorded after counterstaining with TO-PRO3 (nuclei marker), and cardiac myocytes were identified by staining with anti-sarcomeric actinin (green). The apoptotic index was determined by the number of TUNEL positive cardiac muscle cells/the total number of nuclei counted ×100% from a total of 60 fields per slide.

### Statistical analyses

Differences between groups were assessed with Student's t-test and one-way ANOVA. Survival was expressed by the Kaplan-Meier method, and survival curves were compared by the log-rank test. All values are expressed as mean ± S.E.M. A value of *P*<0.05 was considered statistically significant.

## Results

### Changes in CaMKIIδ, NCX1 and SERCA proteins following TAC

**Figure 1:**
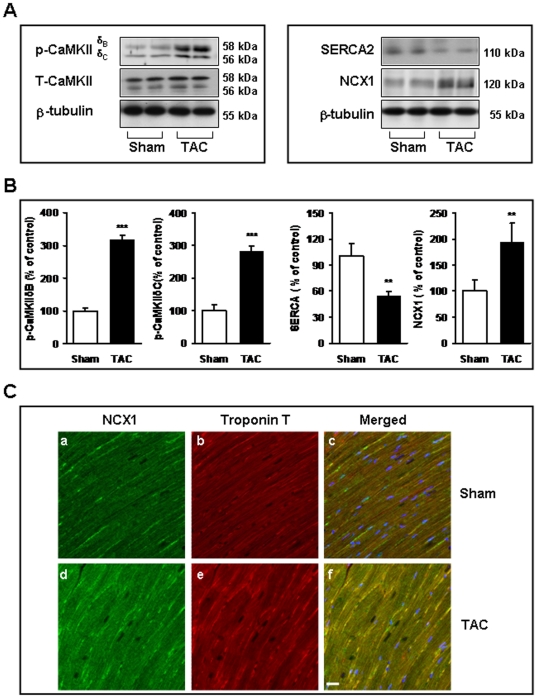
Changes in CaMKIIδ, NCX1 and SERCA2 protein levels following TAC. (**A**) A representative image (upper) and bar graph (lower) showing the results of immunoblotting with anti-CaMKII, anti-NCX1 and anti-SERCA2 antibodies. β-tubulin was used as the loading control. (**B**) Densitometry values were normalized to the average of all sham values, which corresponded to 100% (mean ± SEM, n = 6). ^**^ *P*<0.01, ^***^ *P*<0.001 vs. sham-operated mice. (**C**) The representative confocal images of dual staining with anti-NCX1 (green fluorescence) and anti-troponinT (red fluorescence) antibodies in TAC animals or control heart tissue. DAPI was used as a counterstain, to label the cell nuclei (blue). Scale bar = 20 µm.

We first determined the changes in immunoreactive Ca^2+^-handling proteins by Western blot (Fig. 1A and 1B). Compared with the controls, phosphorylation of both CaMKIIδB and CaMKIIδC were increased (3.1-fold and 2.8-fold, respectively). Alterations in SERCA2 and NCX1 expression are thought to be involved in the pathological process of heart failure [8]–[11]. Consistent with this idea, our results demonstrate that the expression levels of SERCA2 and NCX1 were changed, but in opposite directions, following TAC treatment. SERCA2 protein levels decreased by 40% in the TAC group (Fig. 1A and B), and NCX1 protein levels increased by nearly 2-fold (Fig. 1A and 1B). Moreover, the localization of and changes in NCX1 expression for TAC animals and control hearts were examined by immunohistochemical studies (Fig. 1C). The dual staining of NCX1 and troponinT confirmed that both of these proteins are present at the same location in rat cardiomyocytes, in addition, increased expression of NCX1 was also observed in TAC mice (Fig. 1C).

### Active CaMKIIδB transfection up-regulates NCX1 expression in cultured cardiomyocytes

**Figure 2:**
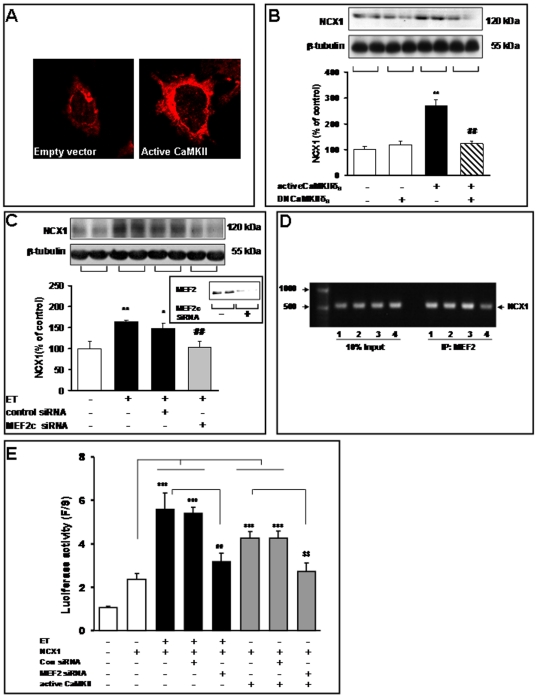
Active CaMKIIδB transfection up-regulates NCX1 levels in cultured cardiomyocytes. After transfection with an empty vector, active CaMKIIδB (T278D), or DNCaMKIIδB (K43E) for 48 h, cardiomyocytes were harvested for immunochemistry and immunoblotting assays. (**A**) Transfection with active CaMKIIδB enriched NCX1 in the membrane, as measured by an immunochemistry assay. (**B**) Detection of NCX1 protein levels in the control and CaMKIIδB-over-expressing cardiomyocytes. A representative image (upper) and bar graph (lower) showing the results of immunoblotting with the anti-NCX1 antibody. β-tubulin was used as the loading control. ^**^ *P*<0.01 vs. cells transfected with an empty vector;^ ##^ *P*<0.01 vs. cells transfected with active CaMKII. (**C**) MEF2c silencing inhibits NCX1 protein expression in ET-treated cardiomyocytes. The cultured cardiomyocytes were transfected with 1 µM of MEF2c-targeting siRNA (siRNA MEF2c) or an siRNA negative control (siRNA control), with or without ET treatment. Cell lysates were collected 48 h after transfection and probed with the MEF2c polyclonal antibody. A representative image and bar graph showing the results of immunoblots for NCX1 protein (upper). The quantitative analysis of protein levels was performed by densitometry of control, siRNA control, and siRNA MEF2c cells (lower). ^*^ *P*<0.05, ^**^ *P*<0.01 vs. cells transfected with an empty vector;^ ##^ *P*<0.01 vs. ET treatments. (**D**) Chromatin immunoprecipitation using anti-MEF2 antibodies. The MEF2/DNA complex was amplified using NCX1 promoter-specific primers. Negative controls included all reagents except DNA. DNA size markers are included on the left. The arrow points to the amplified NCX1 DNA on the right. An input sample was used as a control. 1: con, 2: ET, 3: ET+siRNAcon, 4: ET+siRNA MEF2c. (**E**) Luciferase activity was measured 24 h after co-transfection with NCX1-Luc, siRNAcon, siRNA MEF2c with or without ET (or active CaMKII plasmid) treatment. ^***^ *P*<0.001 vs. cells transfected with NCX1-Luc;^ ##^ *P*<0.01 vs. ET treatments;^ $$^ *P*<0.01 vs. cells transfected with active CaMKII.

To determine the potential role of CaMKIIδB in the protein expression of NCX1, we constructed an expression plasmid encoding for the constitutively active CaMKIIδ3 mutant [active CaMKIIδB (T287D)]. In cardiomyocytes, transfection with active CaMKIIδB led to enriched NCX1 in the membrane, as shown in the immunochemistry assay (Fig. 2A). In addition, as shown in the immunoblot, these cells exhibited a 2.7-fold increase in NCX1 protein expression versus the control cells that were transfected with an empty plasmid (Fig. 2B).

### MEF2 participates in ET-induced NCX1 protein expression

As Ca^2+^/calmodulin signaling has been shown to regulate MEF2/HDAC function, we explored the possibility that CaMKIIδB also plays a role in regulating NCX1 expression *via* the MEF2-mediated pathway. First, we tested the efficacy of MEF2c siRNA to knockdown MEF2C in cultured adult cardiomyocytes in the presence of ET (100 nM). Transfection withMEF2c siRNA (300 ng/ml) almost completely inhibited MEF2 protein expression, as measured by Western blot analysis (Fig. 2C). We then examined the efficacy of MEF2c siRNA on the protein levels of NCX1 in the same context. Transfection with MEF2c siRNA significantly attenuated ET-induced NCX1 expression, whereas the control siRNA had no effect (Fig. 2C).

Furthermore, to determine if MEF2 is bound to the NCX1 promoter, we performed chromatin immunoprecipitation using anti-MEF2 antibodies to collect MEF2/DNA complexes from cultured cardiomyocytes (Fig. 2D). We established that the MEF2 antibody recognized formaldehyde fixed and MNase digested MEF2/DNA complexes and that input DNA was similar in all samples (Fig. 2D). DNA associated with the immunoprecipitation reactions was purified. We found NCX1 promoter DNA was amplified in cultured cardiomyocytes samples incubated with anti-MEF2 antibody, but not in no-antibody control reactions (Fig. 2D). The data suggest MEF2 protein binds to NCX1 promoter DNA.

To further test whether MEF2 can regulate NCX1 expression, cultured cardiomyocytes were co-transfected with a reporter plasmid carrying the human NCX1 promoter driving luciferase expression (NCX1-Luc) and MEF2 siRNA vectors. The ET treatment produced a more than a 5-fold increase in reporter activity, whereas the MEF2 siRNA resulted in significant decreasing (Fig. 2E). Co-transfection with a vector expressing constitutively active CaMKII increased activity of the NCX1-Luc reporter. Conversely, co-transfection with MEF2 siRNA expression vectors decreased active CaMKIIδB (T287D)-mediated activation of the NCX1-Luc reporter (Fig. 2E).

### TAC-induced CaMKIIδB activation and HDAC4 translocation are blocked by a calmodulin antagonist

**Figure 3:**
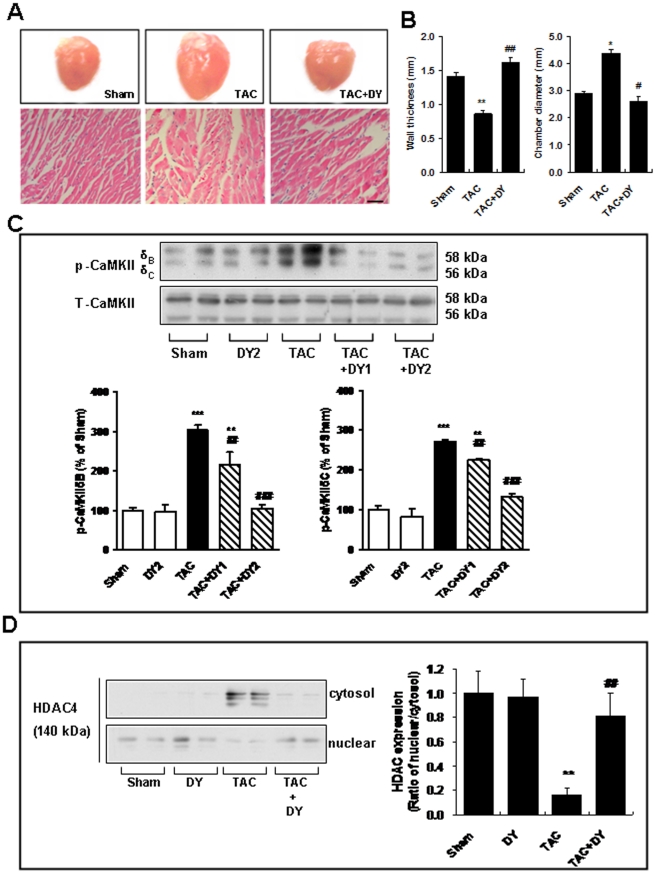
TAC-induced CaMKIIδB activation and CaMKII-HDAC4 complex translocalization are blocked by a calmodulin antagonist. (**A**) Representative hearts (upper) and H&E staining of heart tissues (lower) in mice treated with either a TAC or TAC+ calmodulin antagonist (DY-9836, 20 mg/kg for six weeks). Scale bar = 50 µm. (**B**) The quantitative analyses of wall thickness (upper) and chamber diameter (lower) are shown in the bar graph (mean ± S.E.M., n = 6).^*^ *P*<0.05, ^**^ *P*<0.01 vs. sham-operated group;^ #^ *P*<0.05, ^##^ *P*<0.01 vs. TAC group. (**C**) Top panels: Representative immunoblots from heart tissue lysates of control and TAC mice with or without [DY-9836, 10 and 20 mg/kg (DY1 or DY2)] treatment, assayed with a phospho-CaMKIIδ antibody and the total CaMKIIδ protein. Bottom panels: Quantitative analysis of phospho-CaMKIIδB/C levels was performed by densitometry. Data are expressed as percent values of sham-operated animals (mean ± S.E.M., n = 6). ^**^ *P*<0.01, ^***^ *P*<0.001 vs. sham-operated group;^ ##^ *P*<0.01, ^###^ *P*<0.001 vs. TAC group. (**D**) Immunoblotting analyses of cytosolic HDAC4 (upper) and nuclear HDAC4 (lower) shown markedly decreased levels of HDAC4 nuclear/cytosolic ratio 8 weeks after TAC and indicate that 6 weeks of DY-9836 treatment (20 mg/kg) significantly inhibits the translocation. Right, quantitative analyses are shown in the bar graph as percentage of values of sham operated animals (mean ± S.E.M., n = 6). The nuclear/cytosolic ratio in sham group was expressed as 1. ^**^ *P*<0.01 vs. sham-operated group;^ ##^ *P*<0.01 vs. TAC group.

We previously reported on the cardioprotective effects of calmodulin antagonists, DY-9760e and DY-9836, *in vitro* and *in vivo* through inhibition of Ca^2+^/CaM-dependent enzymes *via* downstream targets during the pathological process of cardiac hypertrophy [19]–[20]. In the present study, our data demonstrate that the ventricular cavity was larger than those in sham animals, and the wall of the ventricle had thinned 8 weeks after TAC (Fig. 3A and 3B). The wall thickness in TAC mice subjected to DY-9836 (20 mg/kg) treatment was significantly decreased when the animals were tested 8 weeks after the final treatment (Fig. 3A and 3B). Additionally, we observed a significant decrease in chamber diameter for DY-9836-treated mice as compared with the TAC animals. To corroborate these results, we also assessed the impact of CaMKIIδB alterations on SR Ca^2+^-handling proteins. As shown in Fig. 1, phosphorylation of CaMKIIδB/C increased significantly during TAC-induced heart failure. Treatment with DY-9836 significantly inhibited TAC-induced phosphorylation of CaMKIIδB/C (Fig. 3C). We also examined whether CaMKII regulated the nuclear-to-cytoplasmic shift of HDAC4, a major CaMKIIδB downstream target. Western blotting was used to quantify changes in the nuclear/cytosolic ratio of HDAC4 (Fig. 3D). Our data showed that HDAC4 and CaMKIIδB dramatically translocated from the nucleus to the cytosol 8 weeks after TAC, whereas HDAC4 and CaMKIIδB were primarily localized to the nucleus in sham animals. Notably, the inhibition of calmodulin partially blocked the TAC-induced nuclear-to-cytoplasmic translocation of HDAC4 (Fig. 3D). Additionaly, we also found that HDAC4 inhibitor trichostatin A decreases ET-induced NCX1 protein expression (Fig.S1).

### Aberrant expression levels of SERCA2 and NCX1 following TAC are both inhibited by a calmodulin antagonist

**Figure 4:**
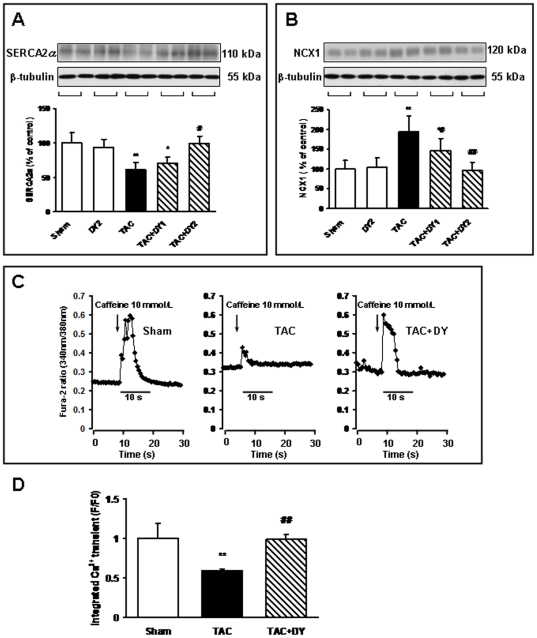
The imbalance of SERCA2 and NCX1 in TAC mice is blocked by a calmodulin antagonist. Immunoblotting was carried out using antibodies that recognized SERCA2 or NCX1 in the indicated groups. Top panels: representative immunoblots of SERCA2 (**A**) and NCX1 (**B**) in the indicated groups. Bottom panels: densitometric analysis of relative SERCA2 (**A**) and NCX1 (**B**) levels. Densitometry values were normalized to the average of all sham values, which corresponded to 100% (mean ± SEM, n = 6). ^*^ *P*<0.05, ^**^ *P*<0.01 vs. sham-operated mice; ^#^ *P*<0.05, ^##^ *P*<0.01 vs. TAC-treated mice. (**C**) Original traces of the current in either sham or TAC adult cardiomyocytes after the application of 10 mmol/L caffeine, either with or without DY-9836 treatment (20 mg/kg) treatment. The arrow indicates the time of caffeine administration. The amplitude of the caffeine-induced Ca^2+^ transient current can be used as an index of SR Ca^2+^ content. (**D**) Mean amplitudes of Ca^2+^ transients in TAC adult cardiomyocytes after treatment either with or without DY-9836 treatment (20 mg/kg). F/F0; fluorescence intensity/background fluorescence levels; ^**^ *P*<0.01 vs. sham cells, ^##^ *P*<0.01 vs. TAC cells.

To gain further insight into the mechanisms of the NCX1/SERCA imbalance in a TAC-induced failing heart, the effects of a calmodulin antagonist on alterations of SERCA2 and NCX1 were examined 8 weeks following TAC treatment. SERCA2 protein levels were decreased in the TAC-treated animals (Fig. 4A), whereas NCX1 protein levels were increased in same context (Fig. 4B). In contrast, treatment with DY-9836 significantly reversed the down-regulation of SERCA2 and the up-regulation of NCX1 (Fig. 4). Additionally, our data show that NCX over-expression induced by endothelin is associated with CaMKIIδB phosphorylation and blocked by a CaMKII inhibitor (KN93) (Fig. S1). In the present study, TAC-induced, dephosphorylated phospholamban (either at Ser16 or Thr17) is tightly associated with SERCA2a down-regulation, which is partially blocked by the pharmacological inhibition of calmodulin (Fig. S2).

### Changes in SR Ca^2+^ content following TAC injury were blocked by calmodulin inhibition

To verify the pathological relevance of CaMKIIδB over-expression and Ca^2+^-handling proteins after TAC treatment, we measured the Ca^2+^ content in the SR by assessing caffeine-induced Ca^2+^ release in cultured adult cardiomyocytes. The Fura-2 ratio at 340/380 nm was not significantly different between the three groups at the baseline. When Ca^2+^ release from the SR was triggered by the application of 10 mM caffeine, transient Ca^2+^ elevation was significantly decreased in the TAC group (Fig. 4C and 4D). As shown in Figures 4C and 4D, the integrative volume of the Ca^2+^ transient in the DY-9836 treated group increased significantly compared with the TAC group. DY-9836 treatment alone (20 mg/kg) did not affect basal Ca^2+^ levels or caffeine-induced Ca^2+^ release (data not shown).

### Effect of calmodulin inhibition on TAC-induced cardiomyocyte apoptosis

**Figure 5:**
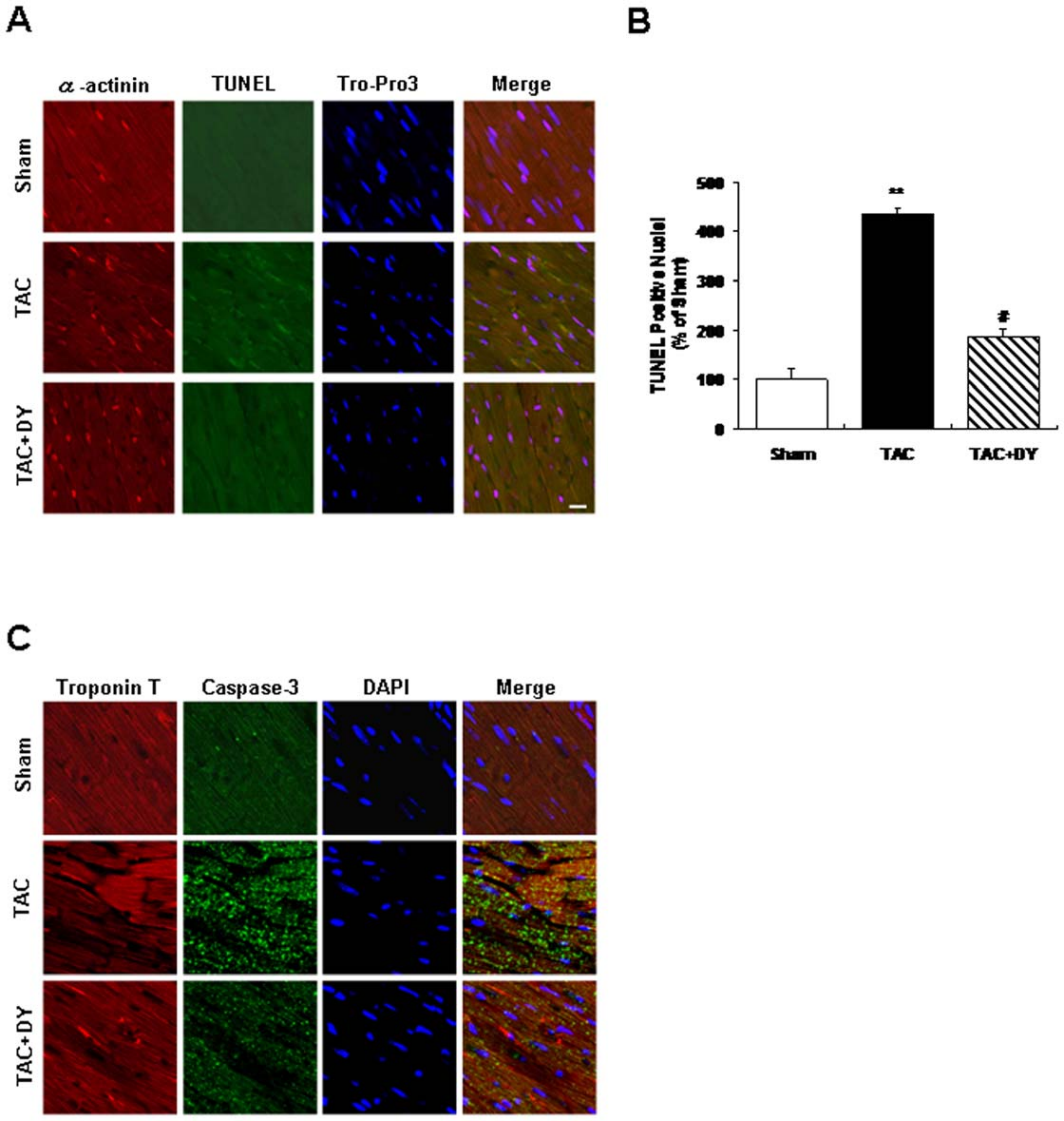
Effect of calmodulin inhibition on TAC-induced cardiomyocyte apoptosis. TUNEL staining was used to assess the number of apoptotic cells. Triple staining was performed: TUNEL (green), TO-PRO3 (blue), α-actinin (red). (**A**) Bar graph shows the increased percentage of TUNEL-positive apoptotic cardiomyocyte nuclei (green fluorescence) 8 weeks after TAC. Scale bar = 20 µm. (**B**) Quantification of TUNEL-positive apoptotic cardiomyocytes in TAC mice with or without DY-9836 (20 mg/kg) treatment. Data are expressed as percentage of sham-operated animals (mean ± SEM, n = 6). ^**^ *P*<0.01 vs. sham; ^##^ *P*<0.01 vs. vehicle. (**C**) Confocal microscopic images of double staining with anti-active caspase-3 (green fluorescence) and anti-troponinT (red fluorescence) antibodies in the heart tissue, indicating that the predominant activation of caspase-3 occurred in TAC mice. The number of cells showing increases in active caspase-3 was significantly reduced by DY-9836 (20 mg/kg) treatment. DAPI was used as a counterstain, to label the cell nuclei (blue). Scale bar = 20 µm.

Cardiac apoptosis contributes to the phenotypic changes associated with heart failure. Changes in apoptosis after 8 week of TAC was detected by TUNEL assay. Images of TUNEL were recorded as described in Materials and methods after counterstaining with TO-PRO3 and alpha-actinin (Fig. 5A). Our results suggest that the number of TUNEL-positive nuclei in TAC animals increased by 4.3-fold versus the control group. In contrast, chronic treatment with DY-9836 (20 mg/kg) significantly decreased TAC-induced apoptosis in mice (Fig. 5A and 5B). Repeated treatment with DY-9836 (20 mg/kg for 6 weeks) alone had no apparent effect on apoptosis in sham-operated animals (data not shown). Confocal microscopic images of double staining with anti-active caspase-3 (green fluorescence) and anti-troponinT (red fluorescence) antibodies in the heart tissue, indicating that the predominant activation of caspase-3 occurred in TAC mice. The appearance of active caspase-3 in the heart tissue was largely abolished by treatment with DY-9836 (Fig. 5C). The same result also observed in Western blot experiment (Fig. S3)

### Effect of calmodulin inhibition on cardiac function and survival rate

**Figure 6:**
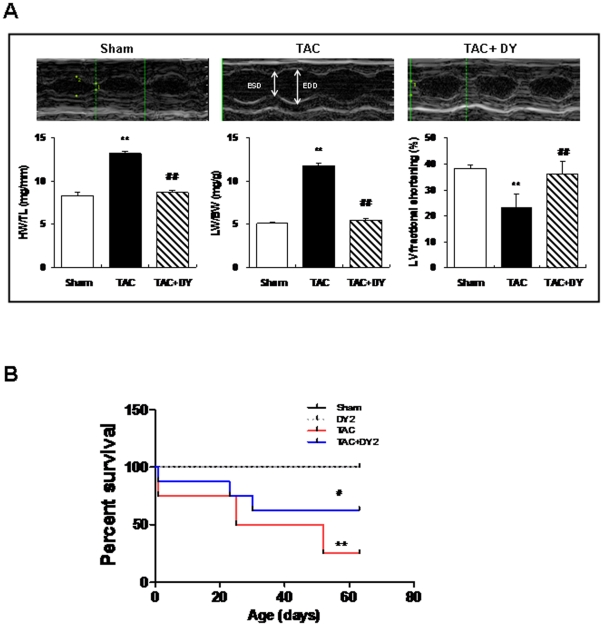
Effect of calmodulin inhibition on cardiac function and survival rate. (**A**) Representative M-mode echocardiographic images were obtained in conscious mice 8 weeks after TAC either with or without DY-9836 (20 mg/kg) treatment. LVIDd: LV end-diastolic internal diameter; LVIDs, LV end-systolic internal diameter; fractional shortening (FS), as follows: %FS = ([LVIDd- LVIDs]/LVIDd)×100. ^**^P,0.01 vs. sham;^##^P,0.01 vs. vehicle. (**B**) The survival of mice after TAC or sham operations was monitored for 80 days. Data are expressed as percentage of sham-operated animals (mean 6 SEM, n = 10). ^**^P,0.01 vs. sham; ^#^P,0.05 vs. vehicle.

Eight weeks after TAC, the treated mice had developed cardiac hypertrophy, as measured by an increased HW/TL ratio compared with the sham group (Fig. 6A). TAC also caused a marked lung edema, demonstrated as a 133% increase in the relative LW/BW ratios. TAC resulted in a significantly greater decrease of fractional shortening than sham group. Notably, TAC-induced changes in the HW/TL and LW/BW ratios and the ejection fraction were inhibited by DY-9836 (20 mg/kg) treatment (Fig. 6A, Table S1). Figure 6B illustrates the dramatic increase in the mortality rate of TAC mice, which was assessed using Kaplan-Meier survival plots. Repeated treatment with DY-9836 (20 mg/kg for 6 weeks) had no effect on sham animals,but markedly decreased mortality to a survival rate of 60% in TAC animals (Log- rank test for mortality = 6.17 (chi square, 1 df), *P*<0.05), as observed at 64 days. The reduced mortality in response to DY-9836 treatment suggests that the maintenance of Ca^2+^ homeostasis protects the heart from cardiac injury under TAC pathological conditions.

## Discussion

We can draw several conclusions from the present study, as follows: 1) aberrant CaMKIIδB expression is associated with a disturbance in SR Ca^2+^ content and an imbalance of NCX1/SERCA2 in TAC-induced heart failure; 2) active CaMKIIδB up-regulates NCX1 protein expression in adult cardiomyocytes *via* HDAC/MEF2-dependent signaling; and 3) the antagonism of calmodulin restores SR Ca^2+^ levels and regulates the imbalance between NCX1 and SERCA2 by inhibiting CaMKIIδB activity, then rescues cardiomyocytes from apoptosis and improves cardiac function.

Numerous disturbances in the function and/or expression of proteins involved in Ca^2+^ handling have been described in the cardiomyocytes of failing hearts. However, the contribution of CaMKIIδ isoforms to transcriptional regulation, Ca^2+^ handling and cardiomyocyte apoptosis, which are factors associated with heart failure, remains unclear. It has been shown that the over-expression of CaMKIIδC phosphorylates Ca^2+^-handling proteins, such as RyR, PLB and L-type Ca^2+^ channel complex, but has no effect on the SERCA and NCX1 protein levels in rabbit cardiomyocytes [21]. Cardiac over-expression of CaMKIIδB causes ventricular dilation and decreased contractile function in transgenic mice. However, the precise intracellular events in CaMKIIδB activation that regulate Ca^2+^-handling protein expression remain to be determined. Therefore, in our present study, we seek to address the role of CaMKIIδB in the TAC-induced imbalance of NCX1 and SERCA2.

We first examined whether CaMKIIδB was involved in the regulation of NCX1 expression. Notably, transfection with active CaMKIIδB induced NCX1 protein up-regulation, as detected by Western blot and immunocytochemical assays. Our results show that an elevation of NCX1 occurs with activation of CaMKIIδB during TAC-induced heart failure, possibly implying a greater reliance on NCX1 to remove cytosolic Ca^2+^ in cardiomyocytes. Additionally, a critical role for HDACs has been proposed in the CaMKII-mediated induction of hypertrophy [22]. In the nucleus, HDACs interact through their N-terminus with MEF2 and repress its transcriptional activity [22]–[24]. Little et al. showed that CaMKIIδB over-expression increases MEF2 transcriptional activity by targeting HDAC4-mediated transcriptional repression in cardiomyocytes [22]. In the present study, we demonstrated that a 3-fold increase in CaMKIIδB activity was accompanied by translocation of HDAC from the nucleus to the cytosol in TAC mice 8 weeks after surgery. This result implies that the activity of CaMKIIδB-phosphorylated HDAC4 and the release of HDAC4-repressed gene expression is a response to TAC, which process to heart failure. Notably, the *Ncx1* promoter contains several consensus sequences for a number of potential DNA-binding factors, including two MEF2 elements at positions −166 and −22 [25]–[26]. Notably, our present results suggest that the translocation of HDAC4 via CaMKIIδB over-expression could affect the transcriptional activation of the NCX1 exchanger. By using MEF2c siRNA transfection, we confirmed that silencing MEF2c induced the NCX1 expression increase in ET-treated adult cardiomyocytes. Moreover, the present CHIP and NCX1-Luc assay data implicated that MEF2 may act in concert with *Ncx1* promoter to shape the magnitude of a NCX1-mediated signal. Thus, we postulate that the CaMKIIδB-induced NCX1 up-regulation is transcriptionally mediated in the TAC model, which may include the translocation of HDACs to the cytosol and MEF2 transcripts, as well as lead to increased expression of proteins directly involved in compensatory Ca^2+^ homeostasis.

In addition, our results demonstrate an increase of NCX1 levels in conjunction both with SERCA down-regulation in the failing heart of TAC animals. SERCA2 transports Ca^2+^ from the cytosol to the SR of cardiomyocytes, thus maintaining the store of releasable Ca^2+^ that is necessary for contraction. We propose that reduced SERCA2 function would be expected to cause immediate, severe myocardial contractile dysfunction. In the present work, we further measured the Ca^2+^ content of the SR by assessing caffeine-induced Ca^2+^ release in TAC mice. Our data demonstrated that the Fura-2 ratio at 340/380 nm was significantly decreased by 40% in the TAC-treated adult cardiomyocytes. Likewise, the reduced SERCA2 function was associated with an increase in NCX1 expression, which could compensate for reduced SR Ca^2+^ cycling by enhancing transsarcolemmal Ca^2+^ cycling by means of NCX during heart failure. Thus, the present data imply that the over-expression of CaMKIIδB might induce an NCX1/SERCA2 imbalance that exacerbates the perturbation of Ca^2+^ homeostasis and causes cardiac dysfunction. Several recent studies have indicated that SERCA2 protein degradation is stimulated by peroxynitrite and prevented by calpain inhibitors [27]. In fact, we reported that Ca^2+^/calmodulin stimulates the degradation of spectrin by calpain in TAC mice [16]. Thus, we propose that a decrease in SERCA2 expression might be due to either calpain activation or peroxynitrite formation in TAC mice.

Although hypertrophy is formed in response to hemodynamic overload, prolonged left ventricle hypertrophy may result in congestive heart failure. The abnormalities in calcium handling and apoptosis of cardiac myocytes have been reported to be involved in the transition from compensated cardiac hypertrophy to decompensated heart failure [28]–[29]. Our results show that antagonism of calmodulin significantly reduced TAC-induced CaMKIIδB over-expression and HDAC translocalization. Treatment with a calmodulin antagonist reversed the TAC-induced, transient Ca^2+^ suppression in mice, concides with inhibitory effect on the apoptosis of cardiac myocytes in vivo and improved survival rate. Therefore, the prolonged calmodulin antagonist treatment might be prevents progression of cardiac hypertrophy to terminal event (heart failure). An enhanced SR-stored Ca^2+^ concentration contributes to the cytosolic Ca^2+^ homeostasis required to rescue cardiomyocytes from apoptosis and recover contractile function. Thus, we propose that the inhibitory effect of the calmodulin antagonist on CaMKIIδB activity contributes to rescue from CaMKII-mediated up-regulation of NCX1 during TAC-induced hypertrophy, which maintains the balance of NCX1/SERCA2 and strengthens the SR Ca^2+^ concentration. It has been reported that CaMKII can directly phosphorylate cardiac SERCA2, resulting in an increase in its enzymatic activity under physiological conditions [30]–[31]. However, our present results do not exclude the possibility that CaMKIIδB-mediated transcriptional cross-talk and compensatory over-expression of NCX1 are subsequently triggered upon inadequate SERCA2 expression. Alternative approaches, such as using animals with genetically modified SERCA2 may be important to further identify the pathogenic process during heart failure.

In summary, our data revealed that the CaMKIIδB-mediated aberrant expression of NCX1 and the imbalance of NCX1/SERCA2 lead to a shift in Ca^2+^ cycling towards more I_Ca_/NCX cycling and a reduction in SR Ca^2+^ uptake and release. This result in a reduction of the SR stored Ca^2+^ concentration during TAC-induced heart failure. The inhibition of CaMKIIδB activity and restoration of the NCX1/SERCA2 balance by a calmodulin antagonist partially mediates its protective effects in cardiomyocytes during heart failure. The present study suggests that targeting calmodulin as well as specifically targeting CaMKIIδB may prove to be an effective approach for the treatment of cardiac hypertrophy and heart failure.

## Supporting Information

Figure S1 **Effect of pharmacological inhibition of CaMKII and HDAC on NCX1 expression.** (**A**) The NCX1 overexpression induced by ET is associated with CaMKIIδB phosphorylation. (**B**) Effect of HDAC inhibitor on NCX1 expression following ET treatment. β-tubulin was used as the loading control. Con, control; ET, endothelin-1; TSA, Trichostatin A.
(TIF)

Figure S2 **Effect of calmodulin antagonist on phosphorylation of PLB in TAC mice.** Representative immunoblots from heart tissue lysates of control and TAC mice with or without [DY-9836, 10 and 20 mg/kg (DY1 or DY2)] treatment, assayed with phospho-PLB antibody (Thr17 or Ser16). ββ-tubulin was used as the loading control.
(TIF)

Figure S3 **The calmodulin antagonist inhibited cleavage of caspase-3 following TAC.** Representative immunoblots of cleaved caspase-3 in sham and TAC mice with or without [DY-9836, 20 mg/kg (DY)] treatment. β-tubulin was used as the loading control.
(TIF)

Table S1 **Echocardiographic measurements in mice with aortic banding.** HR: heart rate; LV: left ventricular; LVIDd: LV end-diastolic internal diameter; LVIDs, LV end-systolic internal diameter; FS: LV fractional shortening. **P<0.01 *vs* Sham; *^#^P*<0.05, *^##^P*<0.01 *vs* TAC.
(DOC)

## References

[1] Yang D, Zhu WZ, Xiao B, Brochet DX, Chen SR (2007). Ca^2+^/calmodulin kinase II-dependent phosphorylation of ryanodine receptors suppresses Ca^2+^ sparks and Ca^2+^ waves in cardiac myocytes.. Circ Res.

[2] Ling H, Zhang T, Pereira L, Means CK, Cheng H (2009). Requirement for Ca^2+^/calmodulin-dependent kinase II in the transition from pressure overload-induced cardiac hypertrophy to heart failure in mice.. J Clin Invest.

[3] Bodi I, Mikala G, Koch SE, Akhter SA, Schwartz A (2005). The L-type calcium channel in the heart: the beat goes on.. J Clin Invest.

[4] Petrashevskaya NN, Bodi I, Rubio M, Molkentin JD, Schwartz A (2002). Cardiac function and electrical remodeling of the calcineurin-overexpressed transgenic mouse.. Cardiovasc Res.

[5] Ramirez MT, Zhao XL, Schulman H, Brown JH (1997). The nuclear deltaB isoform of Ca^2+^/calmodulin-dependent protein kinase II regulates atrial natriuretic factor gene expression in ventricular myocytes.. J Biol Chem.

[6] Zhang T, Johnson EN, Gu Y, Morissette MR, Sah VP (2002). The cardiac-specific nuclear delta(B) isoform of Ca^2+^/calmodulin-dependent protein kinase II induces hypertrophy and dilated cardiomyopathy associated with increased protein phosphatase 2A activity.. J Biol Chem.

[7] Bers DM (2002). Cardiac excitation-contraction coupling.. Nature.

[8] Kent RL, Rozich JD, McCollam PL, McDermott DE, Thacker UF (1993). Rapid expression of the Na^+^-Ca^2+^ exchanger in response to cardiac pressure overload.. Am J Physiol.

[9] Hasenfuss G, Reinecke H, Studer R, Meyer M, Pieske B (1994). Relation between myocardial function and expression of sarcoplasmic reticulum Ca^2+^-ATPase in failing and nonfailing human myocardium.. Circ Res.

[10] Pogwizd SM, Schlotthauer K, Li L, Yuan W, Bers DM (2001). Arrhythmogenesis and contractile dysfunction in heart failure: Roles of sodium-calcium exchange, inward rectifier potassium current, and residual beta-adrenergic responsiveness.. Circ Res.

[11] Hobai IA, O'Rourke B (2001). Decreased sarcoplasmic reticulum calcium content is responsible for defective excitation-contraction coupling in canine heart failure.. Circulation.

[12] Andersson KB, Birkeland JA, Finsen AV, Louch WE, Sjaastad I (2009). Moderate heart dysfunction in mice with inducible cardiomyocyte-specific excision of the Serca2 gene.. J Mol Cell Cardiol.

[13] Anwar A, Schlüter KD, Heger J, Piper HM, Euler G (2008). Enhanced SERCA2A expression improves contractile performance of ventricular cardiomyocytes of rat under adrenergic stimulation.. Pflugers Arch - Eur J Physiol.

[14] Menick DR, Barnes KV, Thacker UF, Dawson MM, McDermott DE (1996). The exchanger and cardiac hypertrophy.. Ann N Y Acad Sci.

[15] Müller JG, Isomatsu Y, Koushik SV, O'Quinn M, Xu L (2002). Cardiac-specific expression and hypertrophic upregulation of the feline Na^+^-Ca^2+^ exchanger gene H1-promoter in a transgenic mouse model.. Circ Res.

[16] Han F, Lu YM, Hasegawa H, Kanai H, Hachimura E (2010). Inhibition of dystrophin breakdown and endothelial nitric-oxide synthase uncoupling accounts for cytoprotection by 3-[2-[4-(3-chloro-2-methylphenyl)-1-piperazinyl]ethyl]-5,6-dimethoxy-1-(4-imidazolylmethyl)-1H-indazole dihydrochloride 3.5 hydrate (DY-9760e) in left ventricular hypertrophied Mice.. J Pharmacol Exp Ther.

[17] Lu YM, Shioda N, Han F, Moriguchi S, Kasahara J (2007). Imbalance between CaM kinase II and calcineurin activities impairs caffeine-induced calcium release in hypertrophic cardiomyocytes.. Biochem Pharmacol.

[18] Day SM, Westfall MV, Fomicheva EV, Hoyer K, Yasuda S (2006). Histidine button engineered into cardiac troponin I protects the ischemic and failing heart.. Nat Med.

[19] Lu YM, Shioda N, Yamamoto Y, Han F, Fukunaga K (2010). Transcriptional upregulation of calcineurin Abeta by endothelin-1 is partially mediated by calcium/calmodulin-dependent protein kinase IIdelta3 in rat cardiomyocytes.. Biochim Biophys Acta.

[20] Lu YM, Han F, Shioda N, Moriguchi S, Shirasaki Y (2009). Phenylephrine-induced cardiomyocyte injury is triggered by superoxide generation through uncoupled endothelial nitric-oxide synthase and ameliorated by3-[2-[4-(3-chloro-2-methylphenyl)-1-piperazinyl]ethyl]-5,6-dimethoxyindazole (DY-9836), a novel calmodulin antagonist.. Mol Pharmacol.

[21] Kohlhaas M, Zhang T, Seidler T, Zibrova D, Dybkova N (2006). Increased sarcoplasmic reticulum calcium leak but unaltered contractility by acute CaMKII overexpression in isolated rabbit cardiac myocytes.. Circ Res.

[22] Little GH, Bai Y, Williams T, Poizat C (2007). Nuclear Calcium/Calmodulin-dependent Protein Kinase II Preferentially Transmits Signals to Histone Deacetylase 4 in Cardiac Cells.. J Biol Chem.

[23] McKinsey TA, Zhang CL, Lu J, Olson EN (2000). Signal-dependent nuclear export of a histone deacetylase regulates muscle differentiation.. Nature.

[24] Youn HD, Grozinger CM, Liu JO (2000). Calcium regulates transcriptional repression of myocyte enhancer factor 2 by histone deacetylase 4.. J Biol Chem.

[25] Cheng G, Hagen TP, Dawson ML, Barnes KV, Menick DR (1999). The role of GATA, CArG, E-box, and a novel element in the regulation of cardiac expression of the Na^+^-Ca^2+^ exchanger gene.. J Biol Chem.

[26] Menick DR, Renaud L, Buchholz A, Müller JG, Zhou H (2007). Regulation of Ncx1 gene expression in the normal and hypertrophic heart.. Ann N Y Acad Sci.

[27] Randriamboavonjy V, Pistrosch F, Bölck B, Schwinger RH, Dixit M (2008). Platelet sarcoplasmic endoplasmic reticulum Ca^2+^-ATPase and mu-calpain activity are altered in type 2 diabetes mellitus and restored by rosiglitazone.. Circulation.

[28] Li XM, Ma YT, Yang YN, Liu F, Chen Bd (2009). Downregulation of survival signalling pathways and increased apoptosis in the transition of pressure overload-induced cardiac hypertrophy to heart failure.. Clin Exp Pharmacol Physiol.

[29] Moorjani N, Ahmad M, Catarino P, Brittin R, Trabzuni D (2006). Activation of apoptotic caspase cascade during the transition to pressure overload-induced heart failure.. J Am Coll Cardiol.

[30] Xu A, Hawkins C, Narayanan N (1993). Phosphorylation and activation of the Ca^2+^-pumping ATPase of cardiac sarcoplasmic reticulum by Ca^2+^/calmodulin-dependent protein kinase.. J Biol Chem.

[31] Toyofuku T, Curotto KK, Narayanan N, MacLennan DH (1994). Identification of Ser38 as the site in cardiac sarcoplasmic reticulum Ca^2+^-ATPase that is phosphorylated by Ca^2+^/calmodulindependent protein kinase.. J Biol Chem.

