# Supplementary figures and images for: CaMKIIδB Mediates Aberrant NCX1 Expression and the Imbalance of NCX1/SERCA in Transverse Aortic Constriction-Induced Failing Heart
Source: PLoS One. 2011 Sep 13;6(9):e24724. doi: 10.1371/journal.pone.0024724 (PMC3172303; doi:10.1371/journal.pone.0024724)

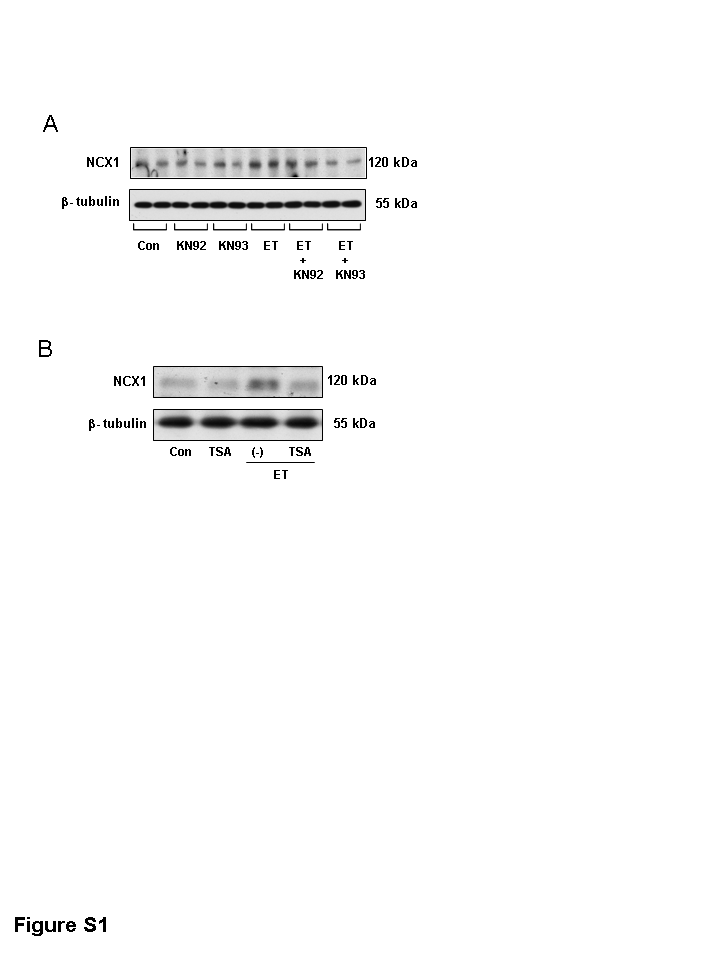

Supplement: Figure S1 — Effect of pharmacological inhibition of CaMKII and HDAC on NCX1 expression. (A) The NCX1 overexpression induced by ET is associated with CaMKIIδB phosphorylation. (B) Effect of HDAC inhibitor on NCX1 expression following ET treatment. β-tubulin was used as the loading control. Con, control; ET, endothelin-1; TSA, Trichostatin A. (TIF) [file pone.0024724.s001.tif]

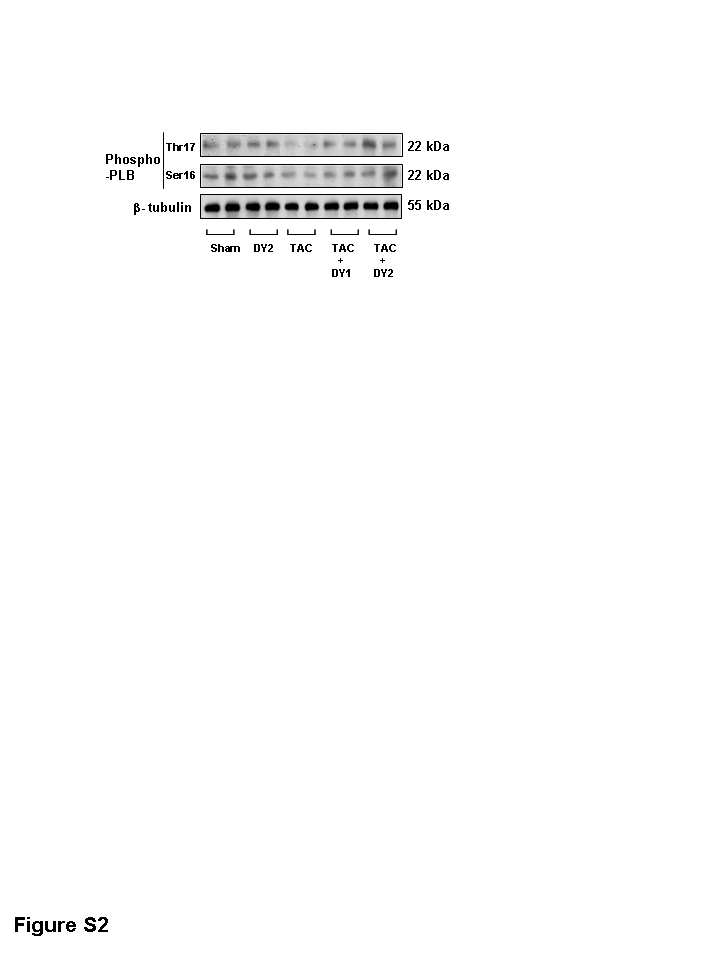

Supplement: Figure S2 — Effect of calmodulin antagonist on phosphorylation of PLB in TAC mice. Representative immunoblots from heart tissue lysates of control and TAC mice with or without [DY-9836, 10 and 20 mg/kg (DY1 or DY2)] treatment, assayed with phospho-PLB antibody (Thr17 or Ser16). ββ-tubulin was used as the loading control. (TIF) [file pone.0024724.s002.tif]

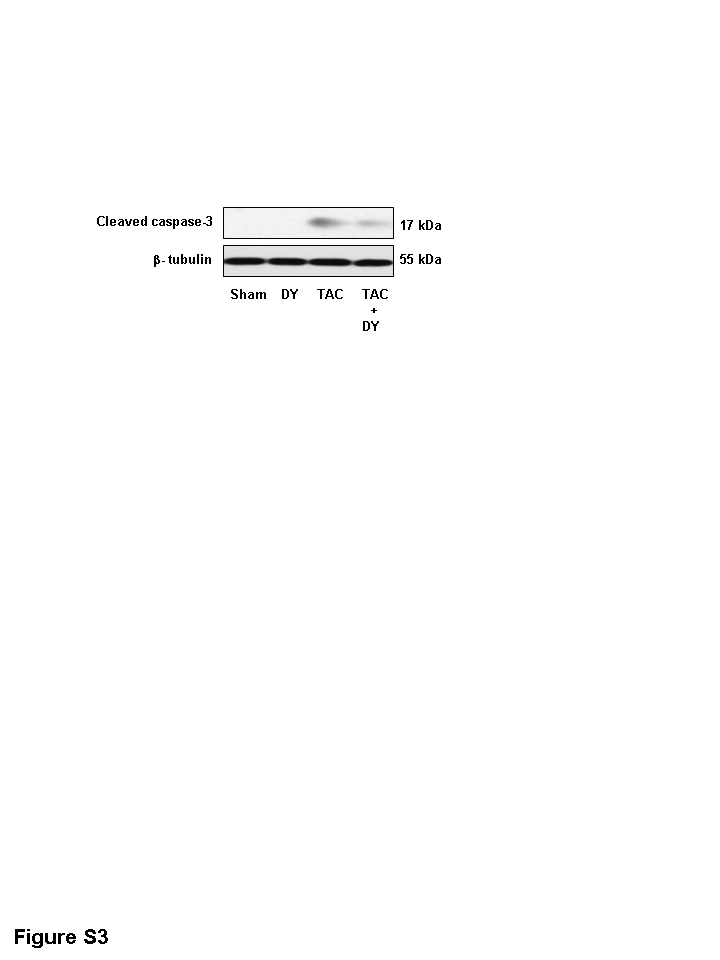

Supplement: Figure S3 — The calmodulin antagonist inhibited cleavage of caspase-3 following TAC. Representative immunoblots of cleaved caspase-3 in sham and TAC mice with or without [DY-9836, 20 mg/kg (DY)] treatment. β-tubulin was used as the loading control. (TIF) [file pone.0024724.s003.tif]
