# Supplementary material for: CaMKIIδB Mediates Aberrant NCX1 Expression and the Imbalance of NCX1/SERCA in Transverse Aortic Constriction-Induced Failing Heart
Source: PLoS One. 2011 Sep 13;6(9):e24724. doi: 10.1371/journal.pone.0024724 (PMC3172303; doi:10.1371/journal.pone.0024724)
Supplement: Table S1 — Echocardiographic measurements in mice with aortic banding. HR: heart rate; LV: left ventricular; LVIDd: LV end-diastolic internal diameter; LVIDs, LV end-systolic internal diameter; FS: LV fractional shortening. **P<0.01 vs Sham; #P<0.05, ##P<0.01 vs TAC. (DOC) [file pone.0024724.s004.doc]

Table 1. Echocardiographic Data

| Parameter | Sham | DY | TAC | TAC+DY |
| --- | --- | --- | --- | --- |
| HR (bpm) | 535±10 | 537±27 | 568±23 | 536±7 |
| LVIDd (mm) | 4.03±0.05 | 4.08±0.22 | 4.28±0.05** | 3.98±0.33^#^ |
| LVIDs (mm) | 2.48±0.05 | 2.50±0.41 | 3.20±0.08** | 2.63±0.22^##^ |
| FS (%) | 38.51±1.23 | 38.89±3.75 | 25.14±1.05** | 33.92±2.78^##^ |

HR: heart rate; LV: left ventricular; LVIDd: LV end-diastolic internal diameter; LVIDs, LV end-systolic internal diameter; FS: LV fractional shortening. ***P*＜0.01 *vs* Sham; *^#^P*＜0.05*,* *^##^P*＜0.01 *vs* TAC.
